# Coital frequency and condom use in monogamous and concurrent sexual relationships in Cape Town, South Africa

**DOI:** 10.7448/IAS.16.1.18034

**Published:** 2013-04-24

**Authors:** Wim Delva, Fei Meng, Roxanne Beauclair, Nele Deprez, Marleen Temmerman, Alex Welte, Niel Hens

**Affiliations:** 1 The South African Department of Science and Technology/National Research Foundation (DST/NRF), Centre of Excellence in Epidemiological Modelling and Analysis (SACEMA), Stellenbosch University, Stellenbosch, South Africa; 2 International Centre for Reproductive Health, Ghent University, Gent, Belgium; 3 Center for Statistics, Hasselt University, Diepenbeek, Belgium; 4 Faculty of Medicine and Health Sciences, Ghent University, Gent, Belgium; 5 Centre for Health Economic Research and Modelling Infectious Diseases, Vaccine and Infectious Disease Institute, University of Antwerp, Wilrijk, Belgium

**Keywords:** coital dilution, condom use, concurrency, HIV, South Africa, sexual behaviour, sex frequency

## Abstract

**Introduction:**

A decreased frequency of unprotected sex during episodes of concurrent relationships may dramatically reduce the role of concurrency in accelerating the spread of HIV. Such a decrease could be the result of coital dilution – the reduction in per-partner coital frequency from additional partners – and/or increased condom use during concurrency. To study the effect of concurrency on the frequency of unprotected sex, we examined sexual behaviour data from three communities with high HIV prevalence around Cape Town, South Africa.

**Methods:**

We conducted a cross-sectional survey from June 2011 to February 2012 using audio computer-assisted self-interviewing to reconstruct one-year sexual histories, with a focus on coital frequency and condom use. Participants were randomly sampled from a previous TB and HIV prevalence survey. Mixed effects logistic and Poisson regression models were fitted to data from 527 sexually active adults reporting on 1210 relationship episodes to evaluate the effect of concurrency status on consistent condom use and coital frequency.

**Results:**

The median of the per-partner weekly average coital frequency was 2 (IQR: 1–3), and consistent condom use was reported for 36% of the relationship episodes. Neither per-partner coital frequency nor consistent condom use changed significantly during episodes of concurrency (aIRR=1.05; 95% confidence interval (CI): 0.99–1.24 and aOR=1.01; 95% CI: 0.38–2.68, respectively). Being male, coloured, having a tertiary education, and having a relationship between 2 weeks and 9 months were associated with higher coital frequencies. Being coloured, and having a relationship lasting for more than 9 months, was associated with inconsistent condom use.

**Conclusions:**

We found no evidence for coital dilution or for increased condom use during concurrent relationship episodes in three communities around Cape Town with high HIV prevalence. Given the low levels of self-reported consistent condom use, our findings suggest that if the frequency of unprotected sex with each of the sexual partners is sustained during concurrent relationships, HIV-positive individuals with concurrent partners may disproportionately contribute to onward HIV transmission.

## Introduction

Concurrent relationships have been defined by the Working Group on Measuring Concurrent Sexual Partnerships of the UNAIDS Reference Group on Estimates, Modelling, and Projections as “overlapping sexual partnerships in which sexual intercourse with one partner occurs between two acts of intercourse with another partner” [1]. The importance of concurrency in driving HIV transmission in hyperendemic settings remains controversial. While some have argued, primarily using modelling studies, that concurrency is a strong facilitator of HIV transmission, or even an essential driver for sustained HIV epidemics [2–4], others have dismissed the concurrency hypothesis, because of perceived flaws in the structure and assumptions of the models used [5] and missing empirical evidence for causal links between levels of concurrency and the local or national HIV prevalence [6–10].

Recently, Sawers *et al*. concluded that the role of concurrency in accelerating the spread of HIV is dramatically reduced by coital dilution – the reduction in per-partner coital frequency that accompanies the acquisition of additional partners [11]. In general, a decreased frequency of unprotected sex during episodes of concurrent relationships would reduce the transmission-facilitating effect of concurrency. Such a decrease could be the result of coital dilution and/or increased condom use during concurrency [12,13].

Despite the large number of sexual behaviour surveys that have investigated condom use, sex frequency and concurrency in settings with high HIV prevalence, few analyses have specifically focused on condom use and sex frequency in concurrent versus monogamous relationship episodes [14]. In this paper, we aim to address this gap by examining self-reported data on coital frequency and condom use during monogamous and concurrent relationship episodes from an egocentric sexual network survey in three communities with high HIV prevalence around Cape Town, South Africa. Besides the concurrency status, we explore associations with a wide range of demographic and relationship characteristics, to identify other, potentially more important factors that influence coital frequency and condom use.

## Methods

### Study design and setting

We conducted a cross-sectional survey (*n*=878) from June 2011 to February 2012 in three urban disadvantaged communities in the greater Cape Town area to study associations between HIV status, sexual connectedness and age-disparity. The study design and protocol is explained in detail elsewhere [15]. In brief, the survey explored one-year sexual histories, with a focus on start and end dates of periods of sexual activity, age differences between sexual partners, sex frequency, condom use and the use of alcohol and recreational drugs. The questionnaire was administered in a safe and confidential mobile interview space, using audio computer-assisted self-interview (ACASI) technology on touch screen computers. ACASI has the benefit of providing privacy to participants and avoids the white coat effect when answering questions about sensitive topics. The ACASI featured a choice of languages and visual feedback of temporal information. All study communities participated in a previous TB/HIV surveillance study, from which HIV test results were anonymously linked to the survey dataset [16]. A list of participants from the TB/HIV surveillance study was generated for each of the three communities, and the names and associated addresses were randomly reordered. Field workers visited the homes of candidate survey participants in the order that they were placed on the list.

Of 1857 people randomly sampled from the TB/HIV surveillance study sampling frame, we were able to find 1115 (60.0% contact rate). For 197 people, the reason for non-retrieval after three attempts is unknown, while for, respectively, 511 and 34, relocation to an unknown new address and death were documented. Eighty-seven candidate participants were excluded, primarily due to visual or physical impairments that rendered participation in the study impossible. Of the remaining 1028, 878 (85.4% response rate) consented to participate.

### Participants and variables

Of the 878 survey respondents, 679 (77.3%) had at least one relationship in the last 12 months. These respondents reported on a total of 1567 relationship episodes from 1128 relationships. Relationship episodes with missing data for coital frequency (*n*=193), condom use (*n*=5), respondent age (*n*=3), partner age (*n*=24) respondent gender (*n*=49), race (*n*=5), completed education level (*n*=1) or employment status (*n*=2) were excluded. Furthermore, episodes were excluded if the respondents did not sleep with their partner in the past year (*n*=14) and if the reported ages of respondents were <18 years or >70 years (*n*=42). In the context of the South African HIV epidemic, the HIV prevalence is considerably higher in black and coloured communities than it is in other racial groups [17]. Our survey was conducted in communities with high HIV prevalence, and consequently, very few people of Indian or white race were included in our sample. Therefore, 19 episodes from three respondents were excluded if the respondents were white, Indian or unknown race, leaving only episodes of black and coloured respondents in the analysis. The term *coloured* refers to a racial category in South Africa, and consists of racially mixed descendants of Europeans, indigenous populations and slaves from South and East Asia.

For up to five main partners and 15 casual partners, participants indicated the periods (episodes) they were in the relationship on a touch screen timeline [15]. A participant could select multiple different time periods for each partner. The dependent variables, frequency of intercourse and condom use, were asked for each episode indicated on the timeline. Periods of a week or longer during which participants indicated not having slept with a particular partner were counted as “breaks” between relationship episodes. For each relationship episode, participants were asked what the weekly average number of sex acts was (0, 1, 2, … 13, 14, 15, >15) and how frequently they used condoms during sexual intercourse (always, sometimes, never). For each round of questions concerning a particular episode, the timing of the episode was highlighted on the touch screen timeline.

**Figure 1:**
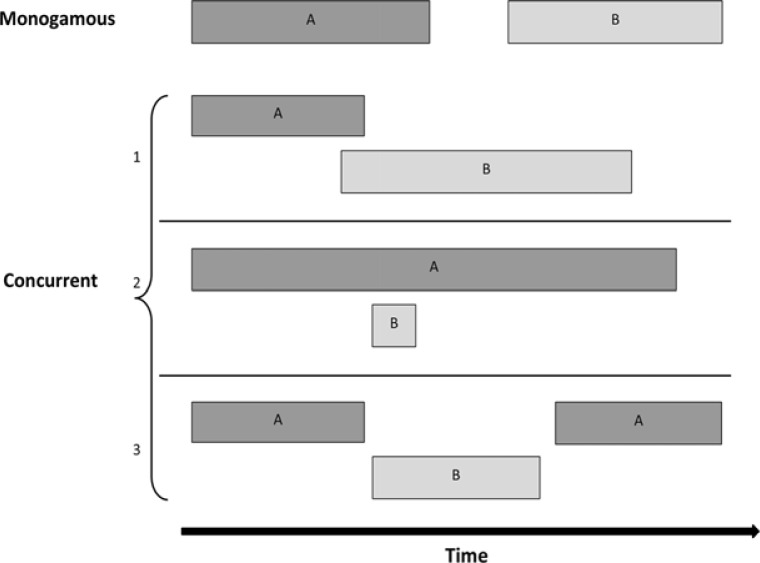
Schematic representation of monogamous and concurrent relationship episodes.

Figure 1 outlines how the concurrency status of each relationship episode was derived from the relationship history time line. Building on the defining characteristic of concurrency that individuals return to a previous partner (A) after having had intercourse with another partner (B), any episode for which this condition was true, was considered concurrent in the primary analysis [1]. Under this definition, as proposed by UNAIDS, 1A, 1B, 2A, 2B and 3B are concurrent episodes. However, this definition may be problematic as it lacks any indication of time scale over which the presence of overlap should be evaluated. Consequently, apparently very different kinds of “overlap” are grouped into the category of concurrent episodes, ranging from a situation in which participants move back and forth between sexual partners multiple times per week for many consecutive weeks, to a situation in which participants alternate between multiple partners, but none of the episodes actually overlap (relationship type 3 in Figure 1). To explore how sensitive our results are to the definition of concurrency, we conducted two parallel analyses. In the first analysis, we applied the literal definition of concurrency according to the UNAIDS reference group (relation episodes 1A, 1B, 2A, 2B and 3B in Figure 1 defined as concurrent). In the second analysis, we only define episodes as concurrent if there is an actual temporal overlap of at least one week (3B in Figure 1 no longer included).

In addition to concurrency status, candidate explanatory variables for the variation in coital frequency and condom use included age (≤25/26–40/>40), race (coloured/black), religion (Christian/other religion/not religious), employment status (employed/unemployed), completed education level (none or primary/secondary/tertiary), age difference between partners (0–4/5–10/>10), relationship duration (≤1 week/2 weeks to 9 months/>9 months) and partner type (casual/main).

### Statistical analysis

First, the coital frequency and condom use data were tabulated and visualized by concurrency status and partner type, and descriptive summary statistics were calculated for all variables under investigation. Next, mixed effects logistic regression and mixed effects Poisson regression models were used to evaluate the effect of concurrency status, on consistent condom use and coital frequency, respectively. These models take into account the correlated nature of the data and variability in the data that stems from both inter- and intra-subject differences in repeated measurements (respondents may report on multiple relationships, which may each consist of multiple relationship episodes) [18]. Backward elimination procedures, based on likelihood ratio tests and Akaike's Information Criterion (AIC), were applied to assess whether employment status, completed education level, religion, age difference between partners, partner type and relationship duration were statistically independent correlates of coital frequency and consistent condom use, after adjusting for concurrency status, race, sex and age.

### Ethical approval

The study was approved by the Stellenbosch University Health Research Ethics Committee (N11/03/093). Written, informed consent was obtained for each respondent prior to administration of the questionnaire.

## Results

After exclusions, 1210 relationship episodes from 828 relationships reported by 527 sexually active respondents were retained. Tables 1 and 2 describe the demographic characteristics of these respondents and key attributes of their reported relationship episodes respectively.

**Table 1:** Individual characteristics of participants in three urban Cape Town communities (aged 18–70 in 2011/2012)

N	n 527	%
Age		
18–25 years	120	22.8
26–40 years	242	45.9
>40 years	165	31.3
Gender		
Male	163	30.9
Female	364	69.1
Race		
Coloured	108	20.5
Black	419	79.5
Education level		
None or primary	162	30.7
Secondary	347	65.8
Tertiary	18	3.4
Employment status		
Employed	404	76.7
Unemployed	123	23.3
Religion		
Christian	350	66.4
Not religious	148	28.1
Other religion	29	5.5
Numbers of partners last year		
1	377	71.5
2	79	15.0
3	37	7.0
>3	27	5.1
Casual partners last year		
Yes	91	17.3
No	436	82.7

**Table 2:** Attributes of relationship episodes from 520 participants in three urban Cape Town communities

N	n 1210	%
Partner type		
Main partner	992	82.0
Casual partner	218	18.0
Concurrency status^a^		
Monogamous	704	58.2
Concurrent	506	41.8
Concurrency status^b^		
Monogamous	719	59.4
Concurrent	491	40.6
Condom use		
Never	382	31.6
Sometimes	391	32.3
Always	437	36.1
Duration		
≤1 week	362	29.9
2 weeks to 9 months	490	40.5
>9 months	358	29.6
Age difference between partners		
<5 years	880	72.7
5–10 years	221	18.3
>10 years	109	9.0
Average Coital frequency per episode		
1	382	29.8
2	378	30.8
3	233	18.2
>3	217	21.2

a UNAIDS defined as any overlapping episode in which sexual intercourse with one partner occurs between two acts of intercourse with another partner. (Relationship episode types 1A, 1B, 2A, 2B, 3B from Figure 1.)

b Our modified definition of concurrency, which excludes relationship episode type 3B from Figure 1.

**Figure 2:**
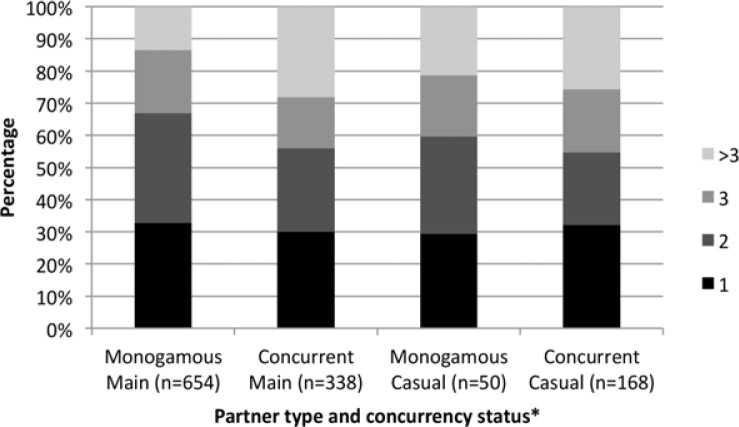
Distribution of coital frequency, by partner type and concurrency status. *Using the UNAIDS definition.

**Figure 3:**
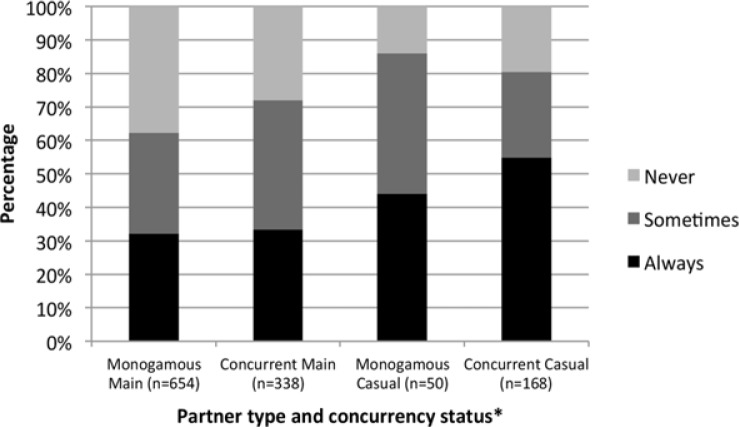
Distribution of condom use, by partner type and concurrency status. *Using the UNAIDS definition.

The majority of respondents were black (80%) and female (69%). While females were clearly represented in higher numbers than males in our survey, the fraction of female respondents in our survey was not very different from that in the sampling frame (62%). Most respondents only reported one sexual partner in the last year (72%), and the vast majority of relationship episodes involved a main partner (82%). Forty-two percent (506/1210) of all episodes were concurrent according to the UNAIDS definition, while 41% (491/1210) were concurrent according to our modified definition. The median of the per-partner average coital frequency was two sex acts per week (IQR: 1–3; mean: 2.5), and consistent condom use (always used condoms) was reported in 36% of episodes. Only 28% (146/527) of the study sample reported consistent condom use in all episodes with all partners of the last year. Figures 2 and 3 depict average weekly coital frequency and condom use reported in each of the 1210 episodes, by concurrency status and partner type.

Figure 2 shows no immediately obvious, stark differences in coital frequencies in monogamous versus concurrent episodes. In the mixed effects regression analysis, presented in Table 3, there was no evidence for concurrency being associated with a lower average coital frequency. Rather, both definitions showed a slight, albeit non-significant, increase in coital frequency during concurrent episodes (UNAIDS definition: aIRR=1.05; 95% confidence interval (CI): 0.99–1.24 and modified definition: aIRR=1.04; 95% CI: 0.98–1.23). Being female (aIRR=0.83; 95% CI: 0.72–0.91), coloured (aIRR=1.34; 95% CI: 1.13–1.48), obtaining a tertiary education (aIRR=1.44; 95% CI: 1.12–1.96), having a relationship of 2 weeks to 9 months in duration (aIRR=1.15; 95% CI: 1.05–1.30) and belonging to an “other” religion (aIRR=1.27; 95% CI: 1.11–1.77) were shown to have a significant association with coital frequency in the model using the UNAIDS definition of concurrency. Using our modified concurrency definition did not qualitatively change these estimates.

**Table 3:** Adjusted incident rate ratios for coital frequency using mixed effects models

	UNAIDS concurrency definition^a^		Our modified concurrency definition^b^	
		
	aIRR	95% CI for aIRR	aIRR	95% CI for aIRR
Concurrent				
No	1.00		1.00	
Yes	1.05	0.99–1.24	1.04	0.98–1.23
Age				
18–25 years	1.00		1.00	
25–40 years	1.03	0.87–1.13	1.03	0.87–1.13
>40 years	0.98	0.81–1.11	0.98	0.81–1.11
Gender				
Male	1.00		1.00	
Female	0.83	0.72–0.91	0.81	0.72–0.91
Race				
Black	1.00		1.00	
Coloured	1.34	1.13–1.48	1.34	1.13–1.48
Partner type				
Main	1.00		1.00	
Casual	1.00	0.94–1.21	1.07	0.94–1.21
Education				
None or primary	1.00		1.00	
Secondary	1.12	0.94–1.22	1.12	0.94–1.22
Tertiary	1.44	1.12–1.96	1.45	1.12–1.96
Duration				
≤1 week	1.00		1.00	
2 weeks to 9 months	1.15	1.05–1.30	1.17	1.05–1.30
>9 months	1.07	0.95–1.22	1.08	0.95–1.23
Religion				
Christian	1.00		1.00	
Not religious	1.03	0.94–1.19	1.04	0.94–1.19
Other	1.27	1.11–1.77	1.28	1.11–1.77

aIRR, adjusted incident rate ratio; CI, confidence interval.

a Defined as any overlapping episode in which sexual intercourse with one partner occurs between two acts of intercourse with another partner. (Relationship episode types 1A, 1B, 2A, 2B, 3B from Figure 1.)

b Excludes relationship episode type 3B from Figure 1.

The condom use outcomes shown in Figure 3 indicate higher consistent condom use in concurrent episodes with casual partners (55%; 44% in monogamous episodes), and similarly low levels of condom use in episodes with main partners, regardless of concurrency status (32–33%). In the mixed effects regression analysis, presented in Table 4, concurrency was not significantly associated with consistent condom use (UNAIDS definition: aOR=1.01; 95% CI: 0.38–2.68 and modified definition: aOR=1.48; 95% CI: 0.58–3.79), but race and relationship duration were. Being coloured (aOR=0.08; 95% CI: 0.01–0.63) and having a relationship duration of more than 9 months (aOR=0.08; 95% CI: 0.03–0.20) were associated with consistent condom use in the model using the UNAIDS definition of concurrency. Similarly to the coital frequency analysis, using our modified concurrency definition did not qualitatively change these estimates. Initial data exploration suggested that partner type was associated with consistent condom use as well, and that there might be effect modification of concurrency status by partner type and by gender. However, partner type could not be included in the final model because of quasi complete separation in the data tables. Furthermore, adding the interaction terms separately, did not improve model fit, and hence these interaction terms were not included in the final model.

**Table 4:** Adjusted odds ratios for consistent condom use using mixed effects models

	UNAIDS concurrency definition^a^		Our modified concurrency definition^b^	
		
	aOR	95% CI for aOR	aOR	95% CI for aOR
Concurrent				
No	1.00		1.00	
Yes	1.01	0.38–2.68	1.48	0.58–3.79
Age				
18–25 years	1.00		1.00	
25–40 years	1.52	0.32–7.19	1.49	0.31–7.24
>40 years	0.87	0.15–4.94	0.90	0.15–5.31
Gender				
Male	1.00		1.00	
Female	1.07	0.27–4.21	1.14	0.28–4.59
Race				
Black	1.00		1.00	
Coloured	0.08	0.01–0.63	0.08	0.01–0.68
Duration				
≤1 week	1.00		1.00	
2 weeks to 9 months	0.46	0.21–1.01	0.42	0.18–0.95
>9 months	0.08	0.03–0.20	0.07	0.03–0.19

aOR, adjusted odds ratio; CI, confidence interval.

a Defined as any overlapping episode in which sexual intercourse with one partner occurs between two acts of intercourse with another partner. (Relationship episode types 1A, 1B, 2A, 2B, 3B from Figure 1).

b Excludes relationship episode type 3B from Figure 1.

## Discussion

Our findings have implications both for the debate around the role of concurrency in the spread of HIV, and more generally for priority setting in HIV prevention. The key factors that determine the role of concurrency in HIV transmission dynamics include: prevalence of concurrent relationships, duration of concurrent episodes, variability of HIV infectiousness with time since infection, connectedness of the entire sexual network and differences in frequencies of HIV exposures (unprotected sex acts) during monogamous versus concurrent episodes [2,10,12,19–21]. Given the large effect of sex frequency and consistent condom use on transmission risk, both coital dilution and increases in consistent condom use could substantially reduce the effect of concurrency on HIV transmission.

This study does not lend support to the coital dilution hypothesis, nor does is it suggest increased condom use during periods of concurrency, after adjusting for confounding variables. Instead, in our study sample of black and coloured respondents from three communities around Cape Town with high HIV prevalence, the coital frequency was higher, but not significantly higher, in concurrent compared to monogamous relationship episodes, regardless of the definition of concurrency. This finding is at odds with the survey findings from sub-Saharan Africa cited by Sawers *et al*. [4,11,22]. It is worth pointing out that Sawers *et al*. make incorrect inferences from Morris *et al*. [4] and Harrison *et al*. [22] by confusing and conflating concurrency status (monogamous versus concurrent) with relationship type (primary versus secondary). We believe the apparent discrepancies between these two studies cited by Sawers *et al*. and ours can be explained by differences in how coital frequency was measured and how concurrency status was assigned. In our survey, participants could indicate multiple relationship episodes with the same partner, with a resolution of one-week time blocks. This allowed us to observe relationships that consisted of multiple, disjointed episodes (14% of main relationships (*n*=92) and 13% of casual relationships (*n*=22)) instead of one continuous time period.

Morris's categorization into “more frequent” and “less frequent” concurrent partners by design creates differences in sex frequency between different sexual partners. However, Morris's analysis does not confirm that coital frequencies are lower in concurrent versus monogamous relationships. Moreover, in the surveys reported by Morris *et al*. participants were asked how many acts of sex they had over the course of the year for “primary” (more frequent) and “secondary” (less frequent) concurrent partnerships, assuming that these partnerships occurred as one continuous episode throughout the year with no gaps. Thus, the survey failed to take into account that some partnerships may have a low cumulative number of sex acts, but consist of one or many short episodes, during which the average coital frequency is high. In the same way, Harrison *et al*. failed to identify relationship episodes and measure coital frequency within each episode. Crucially, they did not restrict analysis of the time since last sex act with the last two sexual partners to respondents who were still in on-going relationships with both these partners. Lack of knowledge of the concurrency status in this analysis of time since last sex act, precludes estimation of the effect of concurrency status on per-partner coital frequency. Sawers *et al*. may therefore have incorrectly inferred coital dilution from larger times since last sex with the second most recent partner.

To our knowledge, this is one of few studies that have attempted to identify behavioural and demographic correlates of coital frequency in concurrent and monogamous relationships [2,23–25]. In our study sample, being coloured, male and having a tertiary education; being in a relationship for a period of 2 weeks to 9 months; and belong to an “other” religion were independent, individual-level predictors of higher coital frequency.

Our crude estimators for consistent condom use in monogamous and concurrent relationship episodes (Figure 3) compare well with related statistics previously reported. In a survey among young black people around Cape Town, 44% of men with a history of concurrency reported consistent condom use [26]. Further, Chopra *et al*. reported more consistent condom use with casual partners than with “steady” partners in a cohort of young Cape Town men of whom 98% reported concurrent relationships in the last three months [27]. Similarly, Maher *et al*. observed that condom use with concurrent partners was more frequent if partnerships were casual instead of “regular”, non-spousal [7].

Results from the mixed effects regression analysis do not provide evidence for increased condom use during concurrency. Other studies, however, have found significant associations between condom use and concurrency status. Of note, Steffenson *et al*. found that in South African men and women aged 15–24, those who had at least one concurrent relationship in the last year (“concurrents”) used condoms less frequently than people in monogamous relationships (“monogamists”) [28]. The discrepancy between their study results and ours might be accounted for by the fact that our analysis was done at the level of relationship episodes, and compares all of the monogamous to all of the concurrent episodes, while adjusting for a range of confounding variables. In contrast, Steffenson *et al*. measured concurrency status at the level of an individual and then compared condom use during only the most recent relationship in “concurrents” and “monogamists”. They, therefore, were not able to accurately determine if concurrent relationships, much less concurrent episodes, are associated with less consistent condom use. Another study, conducted by Kasamba *et al*., explored condom use in spousal and extra-spousal partnerships and found that men who had extra-spousal partnerships were more likely to have ever used condoms with their spouse [29]. Direct comparison with our findings is limited by the fact that they measured “ever having used condoms” and classified relationships into spousal and extra-spousal relationships. We measured “always used a condom” rather than “ever used a condom” because it is a more meaningful indicator of HIV risk aversion.

Implications of our findings for HIV prevention efforts follow primarily from the observation that consistent condom use was generally low, especially in relationships with main partners. Consistent condom use is known to be extremely hard to achieve in long-term, trusting relationships [30], even if they involve transactional sex [31]. Although consistent condom use was more frequently reported with casual partners, as was also seen elsewhere [32,33], there is still a lot of potential for averting HIV transmissions in casual relationships, especially since casual partners may carry a higher burden of sexually transmitted infections, which are known to facilitate HIV transmission [34–37].

Our study has four main limitations. First, in our study, respondents could only report one average weekly coital frequency per episode, regardless of the episode's duration. Consequently, this self-reported average would only be affected minimally, if at all, if coital frequency was temporarily lower during times of concurrency with an episode that overlapped the index episode for a small fraction. Second, left and right censoring of relationships may have led to misclassification of some episodes as monogamous because we had no knowledge of future episodes and episodes that took place more than a year before the survey. Third, the candidate individual-level predictor variables (i.e. religion, employment status, education level, age, sex and race) we explored were asked only at the time of the survey, but used to predict past behaviour (i.e. coital frequency and condom use). Theoretically, these variables may not have stayed constant over the one-year relationship history window. Lastly, our survey data may be subject to bias due to possible dependent errors in reporting concurrency, coital frequency and condom use. We do note, however, that this bias may also have been present in the egocentric survey data that was cited by Sawers *et al*. to support the coital dilution hypothesis. Hence, this bias alone cannot explain the difference between our observations and those cited previously in support of coital dilution.

Despite these limitations, our study had several strengths, which we believe support the accuracy of our results. Rather than face-to-face interviewing, the survey was conducted using ACASI. While comparisons of ACASI and more traditional survey methods have been mixed, several studies that compared ACASI methods with face-to-face interviews in the African context have indicated that participants are more likely to report sexual risk behaviours while using ACASI [38–42]. In addition, we have performed a dedicated analysis of the user-friendliness, privacy and truthfulness of our ACASI instrument. The key conclusion of this paper is that most participants in our survey found the ACASI modality to be acceptable, private, and user-friendly. Moreover, our results indicate less social desirability bias when reporting on multiple, concurrent partners, than in the face-to-face interviews used in Demographic and Health Surveys done in Southern Africa [43]. Furthermore, respondents were asked to place the episodes for each of their relationships in the past year directly on a timeline, progressively from the oldest to the most recent relationship. Thus, the timeline and the episodes of earlier relationships provided visual reference points, which facilitated internal consistency of a respondent's relationship history [44,45]. Finally, our study is unique in that it allowed participants to define their relationships as a series of episodes, which more accurately portrays how people engage in relationships. In reality, relationships are not always continuous: they often have periods of sexual activity and inactivity, and sexual behaviours may not be the same for each new period of a relationship.

## Conclusions

In conclusion, we found no evidence for coital dilution, i.e. for a decreased per-partner sex frequency, or for increased condom use during concurrent relationship episodes in three communities around Cape Town with high HIV prevalence, after adjusting for confounding variables. Instead, concurrency was associated with a slight, borderline-significant (at α=0.05) increase in coital frequency. The main implication of our findings for the concurrency debate is that, if the frequency of unprotected sex with each of the sexual partners is sustained during concurrent relationships, HIV-positive individuals with concurrent partners may disproportionately contribute to onward HIV transmission. Additional analyses from other geographic and epidemiological settings are needed to create a larger body of evidence related to coital frequency and condom use in monogamous and concurrent relationship episodes, and more generally, to deepen our understanding of the determinants of coital frequency and consistent condom use.
